# How should we describe the benefits of palliative radiotherapy?

**DOI:** 10.3747/co.v13i6.108

**Published:** 2006-12

**Authors:** R. Samant, T. Tucker

**Affiliations:** * The Ottawa Hospital Regional Cancer Centre and The Ottawa Hospital, Ottawa, Ontario, Canada; † Faculty of Medicine, University of Ottawa, Ottawa, Ontario, Canada

**Keywords:** Palliative radiotherapy, benefits, treatment responses, description

## Abstract

The value of radiotherapy for palliation is well known to oncologists but not necessarily to other physicians. Using terms such as symptom improvement ratio (sir) and number needed to treat (nnt) rather than traditional response rates might be more appropriate in describing the benefits of palliative radiotherapy to other health care professionals.

## 1. INTRODUCTION

Radiotherapy is a useful treatment modality for cancer patients, and the published literature suggests that approximately 50%–60% can benefit 1–3. However, radiotherapy is known to be underutilized 4–8. Data from Ontario suggest that only 35% of cancer patients actually receive this type of treatment.

Radiotherapy is especially underused for palliation 9. When a treatment can palliate the symptoms of advanced cancer in 50%–80% of patients with relatively little toxicity and inconvenience 10–13, why would it not be used more often?

The reasons for underutilization of radiotherapy are unclear 14–18, but studies have suggested that lack of knowledge among referring physicians about the potential benefits of palliative radiotherapy may play a part. Perhaps radiation oncologists have not been able to describe the value of palliative radiotherapy to health care professionals in an appropriate manner. We therefore decided to review how the clinical effectiveness of palliative radiotherapy is described in the literature, with a view to determining if its description could be improved. We also wanted to determine whether family physicians would find a new method useful for understanding the value of palliative radiotherapy and for discussing it with their patients.

## 2. MATERIALS AND METHODS

### 2.1 Literature Review

We searched the literature using the U.S. National Library of Medicine’s PubMed service to ascertain the various methods used for evaluating and reporting the benefits of palliative radiotherapy. We found more than one thousand studies and, not surprisingly, no uniform way in which the benefits of treatment are reported.

Traditionally in oncology, effectiveness of therapy has been described using the term “response rate” 19–21. Response rates are based on widely reported endpoints for evaluating response to systemic therapy, where “partial response” represents the fraction of patients demonstrating at least 50% reduction in measurable tumour mass for at least 1 month and “complete response” represents the fraction of patients demonstrating complete regression of measurable tumour mass for at least 1 month. These terms refer to a change in tumour volume rather than to how a patient responds symptomatically 22. Whether these terms reflect the clinical benefit of a treatment for patients with advanced and symptomatic cancers is debatable.

In the palliative setting, where symptom relief and quality of life (qol) are more relevant for patients, using a change simply in tumour volume to determine treatment efficacy is not appropriate 23. Symptom response rates should be considered more appropriate for palliative situations. Symptom improvement is a more relevant endpoint for most patients with incurable cancer, even if it is subjective in nature and not always uniformly reported. Consequently, describing the likelihood of symptom improvement after radiation could be more meaningful than using the traditional response rates would be. For example, improvements in bone pain following radiotherapy are often quoted as being 70%–90%, and these improvements have been clearly documented using effective, objective, and validated methods 24,25.

The published literature confirms that palliative radiotherapy can be used for numerous malignancies and in many clinical situations 14–18,24–31. It is especially useful for tumour-mass effects. Table I shows typical scenarios for which palliative radiotherapy is routinely used, along with examples of commonly reported rates of symptom improvement. Admittedly, the methods for quantitatively determining clinically significant responses to treatment in many situations—hemoptysis, dysphagia, spinal cord compression, and brain metastases, among others—remain relatively crude and quite subjective. They represent an area for further investigation.

Data about qol, which is considered key to determining the value of palliative therapies 23,32, has not in the past been routinely evaluated following radio-therapy 33,34. More recent studies have been evaluating the effects of palliative radiotherapy on qol, and the results are now starting to be reported 35–38. However, the approaches to reporting qol changes are widely varied, and no consensus has arisen about the most appropriate or useful ways to make such reports 32,39–41. Also, it can be difficult to interpret qol results from studies and to explain them to patients 39. How best to summarize qol effects resulting from palliative radiotherapy therefore remains problematic.

### 2.2 Symptom Improvement Ratio

Using the information gathered from the literature, we discussed various approaches for describing the clinical benefits of palliative radiotherapy. Our goal was a method that would be both simple to understand and of practical value to referring physicians and their patients. It also had to be consistent with the goals of palliative care.

To describe the symptomatic improvement or clinically significant responses achieved with palliative radiotherapy, we suggest the symptom improvement ratio (sir) concept. Whereas the term “clinically significant response rate” may be confusing to some and difficult to distinguish from the commonly used “response rate,” the sir clearly describes the goal of palliative radiotherapy, which is alleviation of suffering through symptom improvement.

The sir represents, in absolute terms, the proportion of patients who, following treatment, have a clearly documented improvement in one or more predetermined objective and evaluable symptoms. The sir can be obtained by reviewing the published literature from prospective randomized and nonrandomized trials of palliative radiotherapy. It represents the average or approximate rate of clinically significant improvement in a specific symptom such as pain or hemoptysis. It could serve as a practical means of explaining the likelihood of symptom improvement following palliative radiotherapy in a variety of situations. For example, a patient with lung cancer and hemoptysis could be told to expect an 80% chance of resolution after radiotherapy.

The reciprocal of the sir is the number needed to treat (nnt). The nnt is the inverse of the absolute benefit of a treatment intervention; it represents the number of individuals with a particular condition that would have to be treated for the desired benefit to be seen or the unwanted outcome to be prevented in one individual 42–44. In general, the lower the nnt, the larger the magnitude of benefit from the treatment intervention.

Table ii gives examples of estimated sir values with their associated nnt values for typical situations in which palliative radiotherapy has been evaluated. The nnt has become an important way to express the benefit of an active treatment to physicians and patients, and it is also being used to assess the efficacy of radiotherapy 45,46. It has gained wide popularity and acceptance as an important aid to medical decision-making 47.

### 2.3 Pilot Study

We developed a 1-page pilot questionnaire about the usefulness of the terms “response rate,” “sir,” and “nnt.” This questionnaire was given to Canadian family physicians attending a local palliative care and oncology update meeting in Ontario. Of 42 family physicians attending the meeting, 20 (48%) completed the questionnaire. All respondents participated in the care of cancer patients, but they had varying degrees of experience.

Of the 20 respondents, 55% found the term “response rate” confusing, and 70% thought that patients would not understand the term. The term “sir” was thought by 60% possibly to be useful to health care professionals and patients. Approximately 50% thought that the sir was easier to understand than the term “response rate,” although 25% were unsure. Nearly all (85%) thought that the nnt was useful to determine the value of a treatment, and 80% reported routinely considering the nnt of a treatment for their patients. A majority (65%) felt that having an nnt value for palliative radiotherapy would help them to determine whether a patient should be referred for treatment.

## 3. DISCUSSION

The published literature clearly shows the significant clinical benefits and relatively low morbidity derived from palliative radiotherapy 10–13, and so it is somewhat puzzling that radiotherapy is not used more often. Few other medical interventions have an nnt that is consistently less than 2 42,47. The fact that radiotherapy is extremely useful for palliation and yet considerably underutilized suggests that lack of adequate knowledge among referring physicians and other health care professionals is at least partly contributing to the situation 16,17. Confusion regarding terms such as “response rate,” which has various interpretations, may also be a factor. The idea of symptom improvement is more relevant and clear to patients than response rate, and this greater relevance could lead to a better understanding of the value of palliative radiotherapy. For example, using the sir for bone metastases, a physician would be able to say to a patient, “There is an 80% likelihood that your bone pain will get better after some radiation.”

We believe that better education regarding the value of radiotherapy is required for physicians and other health care professionals dealing with cancer patients. These caregivers need to understand exactly how effective radiotherapy can be in many common situations. Using terms such as the sir is a step towards this goal. The sir is a very simple way of describing the clinical benefit of radiotherapy or the likely improvement afterwards in a particular palliative situation. It can also be used in conjunction with terms such as the nnt, to which physicians are already accustomed.

We realize that the sir is not an all-inclusive term when it comes to describing palliative radiotherapy. For example, it does not express the magnitude of the benefit, the duration of symptom improvement, or the toxicity. We also recognize that many studies evaluating symptom improvement after palliative radiotherapy have used subjective methods rather than validated tools to determine efficacy, and work therefore remains to be done to better document and describe various sirs. However, the sir can give a straightforward estimate of benefit, and the concept is easier for physicians and patients to understand than are many of the various qol scales and values that are being reported.

Our pilot study has an admittedly small sample size, but the results suggest that the sir might be helpful is describing the value of palliative radiotherapy and less confusing to physicians and their patients than is the term “response rate.” With use of the sir, physicians would have a better idea of the role of radiotherapy in various situations faced by patients with advanced cancer.

As the sir relates to palliative radiotherapy, it can also be used as another simple measure of treatment efficacy. The term is already familiar to many physicians. Other authors have also discussed the use of sir for evaluating the effectiveness of radiotherapy 45,46. We believe that, if physicians and other health care professionals have a better understanding of palliative radiotherapy through terms such as the sir and nnt, they will be more likely to refer their patients for treatment. This outcome remains to be proven, however.

## 4. CONCLUSIONS

The concept of the sir is straightforward and easily applied in describing the benefits of palliative radiotherapy in various clinical situations. It could be used by referring physicians and their patients in conjunction with the nnt as an aid to decision-making in the setting of supportive care in cancer.

## Figures and Tables

**TABLE I:** Common oncologic conditions for which palliative radiotherapy is used and the associated published rates of symptom improvement 14–18,24–31

*Condition*	*Rates of symptom improvement*
Metastatic bone pain	
Partial relief	70%–94%
Complete relief	28%–80%
Hemoptysis	72%–86%
Chest pain (lung cancer)	59%–86%
Dyspnea (lung cancer)	41%–66%
Cough (lung cancer)	48%–66%
Dysphagia	61%–65%
Superior vena cava obstruction	60%–90%
Brain metastases	50%–70%
Spinal cord compression	64%–73%

**TABLE II:** Examples of symptom improvement ratio (sir) after palliative radiotherapy and corresponding number needed to treat (nnt) values for some common situations

*Condition*	*Approximate SIR*	*NNT (from SIR value)*
Painful bone metastases		
Partial relief	0.8	1.25
Complete relief	0.4	2.5
Hemoptysis	0.8	1.25
Chest pain (lung cancer)	0.7	1.43
Superior vena cava syndrome	0.75	1.3
Brain metastases	0.6	1.67
Spinal cord compression	0.65	1.5
